# Impact of Sucrose as Osmolyte on Molecular Dynamics of Mouse Acetylcholinesterase

**DOI:** 10.3390/biom10121664

**Published:** 2020-12-12

**Authors:** Sofya V. Lushchekina, Gaetan Inidjel, Nicolas Martinez, Patrick Masson, Marie Trovaslet-Leroy, Florian Nachon, Michael Marek Koza, Tilo Seydel, Judith Peters

**Affiliations:** 1 N.M. Emanuel Institute of Biochemical Physics, Russian Academy of Sciences, 119334 Moscow, Russia; sofya.lushchekina@gmail.com; 2 Institut Laue Langevin, 38000 Grenoble, France; gaetan.inidjel@laposte.net (G.I.); nico100885@hotmail.com (N.M.); koza@ill.fr (M.M.K.); seydel@ill.fr (T.S.); 3 Université Grenoble Alpes, UFR PhITEM, LiPhy, CNRS, 38000 Grenoble, France; 4 Neuropharmacology Laboratory, Kazan Federal University, Kremlevskaya str 18, 480002 Kazan, Russia; pym.masson@free.fr; 5 Institut de Recherche Biomédicale des Armées, 91223 Brétigny sur Orge, France; mtrovaslet@hotmail.com (M.T.-L.); florian.nachon@def.gouv.fr (F.N.)

**Keywords:** cholinesterase, osmotic stress, neutron scattering, molecular dynamics, MD simulations

## Abstract

The enzyme model, mouse acetylcholinesterase, which exhibits its active site at the bottom of a narrow gorge, was investigated in the presence of different concentrations of sucrose to shed light on the protein and water dynamics in cholinesterases. The study was conducted by incoherent neutron scattering, giving access to molecular dynamics within the time scale of sub-nano to nanoseconds, in comparison with molecular dynamics simulations. With increasing sucrose concentration, we found non-linear effects, e.g., first a decrease in the dynamics at 5 wt% followed by a gain at 10 wt% sucrose. Direct comparisons with simulations permitted us to understand the following findings: at 5 wt%, sugar molecules interact with the protein surface through water molecules and damp the motions to reduce the overall protein mobility, although the motions inside the gorge are enhanced due to water depletion. When going to 10 wt% of sucrose, some water molecules at the protein surface are replaced by sugar molecules. By penetrating the protein surface, they disrupt some of the intra-protein contacts, and induce new ones, creating new pathways for correlated motions, and therefore, increasing the dynamics. This exhaustive study allowed for an explanation of the detail interactions leading to the observed non-linear behavior.

## 1. Introduction

Investigations of the impact of osmotic stress on biological systems can play major role in the case of extreme environments, for instance, high salinity in oceans, the Dead Sea, and salty lakes. Osmotic stress may also be responsible for physio-pathological processes and the iatrogenic effects of injected drugs. High concentrations of osmolytes in protein solutions have important biotechnological and pharmaceutical applications. Moreover, osmotic pressure can serve as a tool to study particular parts of proteins, if access to pores or other confined regions is not possible otherwise. Such a strategy was chosen in the present case to shed light on the dynamics of the gorge of mouse acetylcholinesterase (mAChE). Acetylcholinesterase (AChE) is a key enzyme in the nervous system that terminates neurotransmission at central cholinergic synapses and at neuromuscular junctions by hydrolyzing the neurotransmitter’s acetylcholine. The structures of cholinesterases (ChE) from different species are very close to each other with, for instance, identity scores between human AChE (hAChE) and mAChE of more than 82%. Due to the interest of cholinesterases as biopharmaceuticals for pre- and post-exposure treatments of organophosphorus poisoning [1], it is important to investigate the effect of sucrose as a protein structure protectant on the molecular dynamics of a model cholinesterase.

The first solved crystalline structure of a cholinesterase was that of *Torpedo californica* AChE (TcAChE) [2]. It showed that the catalytic active site is located at the bottom of a deep (20 Å) and narrow gorge (diameter of about 5 Å at the narrowest point, called “bottle neck”). This means that the substrate hydrolysis takes place in a mostly closed space virtually isolated from the bulk solvent. Along the passageway, the channel is so narrow that substrates would have no access to the active site if the enzyme were too rigid. However, Tai et al. [3] demonstrated. by means of molecular dynamics (MD) simulations, that half of the atoms in mAChE are participating in the so-called breathing mode that is periodically opening the gorge. The groups of McCammon [4,5,6] and Xu [7,8] undertook simulations of mAChE over 1 ns and 20 ns, respectively. The authors analyzed the dynamics and the possible opening of a so-called backdoor and other possible side channels in detail to shed light on movements in the gorge. Water molecules and co-solutes participate as well in the transport of the substrate to the enzyme catalytic center. Finally, water is the co-substrate of AChE-catalyzed hydrolysis of acetylcholine and other esters.

The experimental study of the motions in and out of the gorge is feasible by means of incoherent neutron scattering, which is sensitive to movements mainly of the nuclei of hydrogen (H) atoms, as the incoherent neutron scattering cross section of H is much higher than for any other nucleus. However, neutron scattering does not allow for distinguishing individual particles, which means that one observes the averaged motion of the H nuclei, and of molecular subgroups to which they are bound, assuming that they are representative for the dynamics inside the protein. In principle, incoherent neutron scattering would thus highlight parts of the sample by exchanging H against its isotope deuterium (D) and follow the dynamics of H belonging to the solution only, if the protein were deuterated. However, as so far it was not possible to deuterate ChEs, such an approach had to be excluded in this instance. Instead, the idea was to expose mAChE to sucrose unplugging all molecules from the gorge, to compare such sample to a native one in pure water and to extract information about motions of the solvent molecules only.

mAChE is a very fast enzyme, especially for a serine hydrolase, functioning at a rate approaching that of a diffusion-controlled reaction. The high speed of the enzyme is essential for the rapid functioning of cholinergic synapses, with a turnover of 10^3^–10^4^ s^−1^. Therefore, we postulate that water dynamics within the gorge could be speeded up to facilitate transport of the substrate, eventually even beyond the normal diffusion rate of bulk water molecules. Such effects were observed, for instance, when water molecules were confined within a hydrophobic matrix [9]. The gorge could certainly be assumed to resemble such a confined environment.

Recently, we measured the dynamics of hAChE under high hydrostatic pressure, and our data suggests that this enzyme enters an intermediate folded state at 1.5 kbar, before being fully denatured at 3 kbar [10]. Osmotic pressure is believed to have different effects on reactions, as already discussed theoretically [11,12] and proven for butyrylcholinesterase (BChE) [13]. By using water-cosolvent mixtures, the enzyme hydration state can be modified, and thus, also the molecular dynamics and the transfer velocity of ligands and substrates. An osmolyte added to the solvent can either modify the viscosity only, as for instance glycerol (molar mass of 92 g/mol), or have a real osmotic effect, as for instance the bigger molecule of sucrose (molar mass of 342.3 g/mol). Sucrose is not able to penetrate the active site gorge of AChE and plays therefore the role of a semi permeable membrane by pumping the water molecules from inside the enzyme. Comparing the dynamics of AChE in the presence or absence of sucrose may hence provide information about the dynamics of water inside the gorge. Such information is extremely difficult to get by any other technique. Both effects, viscosity and osmotic pressure, can possibly have a great influence on the internal dynamics as well as the global diffusion of the protein and induce changes in enzymatic activity. Experiments with enzymes in the presence of an osmolyte would allow us to complete our understanding of how the external conditions affect AChE dynamics and the water dynamics inside the gorge.

To better understand our results, we have undertaken molecular dynamics (MD) simulations of the same system at various sucrose concentrations. MD simulations give access to time scales very similar to those probed by incoherent neutron scattering, which allows us to undertake a direct comparison of dynamics and gain a better understanding of mechanisms at the atomic level.

## 2. Materials and Methods

### 2.1. Sample Preparation

Full cDNA of mAChE were inserted into pGS vector and expressed in Chinese hamster ovary cells (CHO-K1 cells). The transfected cells were selected and maintained in BioWhittaker^®^ UltracultureTM medium (Lonza, Belgium) containing methionine sulfoximide (50 μM). The enzymes secreted into the culture medium, were purified using a procainamide affinity chromatography and were concentrated as previously described [14]. The gene produces a monomer, but the enzyme dimerizes spontaneously at the concentrations used here without inter-monomeric covalent binding. In its monomeric form, mAChE has a molecular mass of 66 kDa.

Activity measurements were carried out at 25 °C according to Ellman method [15] using 1 mM acetylthiocholine (ATC) as the substrate and 0.5 mM 5-5′-dithio-bis (2-nitrobenzoic acid) (DTNB) in 0.1 M phosphate buffer pH 7.0.

The preparation of hAChE was previously described in [14]. mAChE was prepared exactly according to the same protocol. Briefly, mAChE was first dialyzed against 25 mM ammonium acetate dissolved in D_2_O (pD = 7.0) and then freeze dried (12 h) at 220 K under vacuum. This salt free protein powder, placed in an appropriated aluminum sample container, was dried for 12 h at atmospheric pressure over P_2_O_5_ and weighed. This measured weight was the sample dry weight (h = 0 g D_2_O/g dry powder, denoted by g/g). For neutron experiments, the sample was hydrated by vapor exchange over D_2_O, at ambient temperature, in a desiccator, until a final water content of about 0.4 g/g was achieved. To verify that no loss of material had occurred, and that the hydration state was the same, the samples were weighed before and after the neutron scattering experiments and no losses were detected.

We prepared four samples of mAChE with 0%, 5%, 10%, and 15% of deuterated sucrose and hydrated them over D_2_O. We have chosen deuterated sucrose from Omicron Biochemicals, Inc., USA, to avoid a significant contribution from its H atoms to the incoherent neutron scattering signal. For the same reason, we used D_2_O for hydration instead of H_2_O. The proportions were chosen so that the weight of sucrose corresponded to the (*w*/*w*)% of mass of sucrose per mass of (sample + D_2_O) (see Table 1). We also prepared the buffer in D_2_O with 0 and 15 (*w*/*w*)% sucrose, corresponding to the extreme values and interpolating the intermediate values, as this is needed for data correction.

### 2.2. Elastic Incoherent Neutron Scattering

The protein was probed elastically and quasi-elastically by incoherent neutron scattering. We used three different spectrometers at the Institut Laue Langevin (ILL), France: the cold neutron time-of-flight spectrometer IN6 [16], the thermal backscattering spectrometer IN13 [17] and the high-resolution backscattering IN16 [18]. The spectrometers give access to molecular motions within the time windows of approximately 20 ps, 100 ps, and 1 ns, respectively. The experiments were conducted consecutively using the same samples.

The elastic incoherent neutron scattering (EINS) intensity is given within the Gaussian approximation by the dynamic structure factor at zero energy exchange:

(1) $$S_{el}\left(Q,\omega =0\pm \Delta E\right)\approx S_{0}\;\exp\left(-\frac{1}{3}\langle u^{2}\rangle Q^{2}\right)$$

where <*u*^2^> is the average atomic mean square displacement (MSD). *ω* and *Q* are the exchanged energy and momentum in units of ħ, respectively, and Δ*E* is the half width half maximum (HWHM) of the instrumental energy resolution related to the time window through Heisenberg’s uncertainty principle. The Gaussian approximation supposes that any atom can only undergo harmonic isotropic motions around its equilibrium position. The average MSD is related to the flexibility of the sample at a given temperature. For *Q* → 0, the approximation is strictly valid, and it holds up to <*u*^2^> *Q*^2^ ≈ 1. The MSD can then be obtained for each temperature by the slope of the semi-logarithmic plot of the incoherent scattering function through:

(2) $$\langle u^{2}\rangle \approx -3\frac{dInS_{el}\left(Q,\omega =0\pm \Delta E\right)}{dQ^{2}}$$

The transmission values were measured on IN13 and were all above 0.9. Consequently, multiple scattering effects should not be taken into consideration for the data treatment. In order to obtain the scattered intensities of the sample, scattering from the empty sample holder and the buffer were subtracted. For the intermediate sucrose concentrations, we interpolated the curves of the buffer measured at 0 and 15 wt%. The data were normalized to a 2 mm thick vanadium slab, a totally incoherent scatterer, providing the relative detector efficiency and the instrumental resolution. Absorption correction was based on the correction formula of Paalman–Pings [19]. The complete data reduction was carried out using the LAMP software available at ILL [20].

Quasi-elastic neutron scattering (QENS) is the part of scattering where small amounts of energy are exchanged between the neutrons and the sample, giving rise to a broadening of the elastic peak. The quasi-elastic structure factor

(3) $$S\left(Q,\omega \right)=e^{-\frac{1}{3}\langle u^{2}\rangle Q^{2}}\left[A_{0}\left(Q\right)\delta \left(\omega \right)+\underset{i}{\sum }A_{i}\left(Q\right)L\left(\Gamma_{i},\;\omega \right)\right]$$

contains the Debye–Waller factor $e^{-\frac{1}{3}\langle u^{2}\rangle Q^{2}}$, an elastic part, proportional to a delta-function in ω and representing the particles whose motions are not resolved within the instrumental setup. Furthermore, it includes a sum over Lorentzians, L, which describe different motional contributions. In theory, the sum can be extended up to an infinite number of contributions, but in practice a few Lorentzian curves are sufficient to describe the data reasonably well without over-fitting them. Here, we used two Lorentzian curves for the buffer and two Lorentzians for the sample, as more Lorentzians did not improve the fit quality. The amplitudes *A*_0_(*Q*) and *A_i_*(*Q*) depend on the momentum transfer *Q*. One can demonstrate [21] that the elastic incoherent structure factor (EISF) *A*_0_(*Q*) is given by the elastic intensity divided by the sum of elastic and quasi-elastic intensities at t → ∞. It vanishes except for *Q* = 0 if only long-range diffusive motions were present, and it is indicative of the geometry of the diffusional process otherwise. For the rotational diffusion of a particle, the EISF is unity at *Q* = 0 and falls to a minimum at a *Q* value which is inversely related to the radius of gyration of the rotating particle.

For data analysis, the structure factor must be convoluted with the instrumental energy resolution, which can be mimicked by a vanadium measurement:

(4) $$S_{exp}\left(Q,\omega \right)=S\left(Q,\omega \right)⨷S_{res}\left(Q,\omega \right)$$

When fitting the structure factor, it is possible to separate elastic and quasi-elastic parts. The Lorentzians are characterized by parameters of the motions, which can be extracted from their HWHM Γ_*i*_. Indeed, the form of the HWHM as function of *Q*^2^ informs about the movements present in the sample. A pure translational diffusion in an unrestricted homogeneous medium corresponds to Brownian motion [22], whose characteristics are a linear behavior of the HWHM in *Q*^2^. The structure factor in this case reads:

(5) $$S_{B}\left(Q,\omega \right)=\frac{1}{\pi }\frac{D_{T}Q^{2}}{\omega^{2}+{\left(D_{T}Q^{2}\right)}^{2}}$$

where *D_T_* represents the translational diffusion coefficient and Γ*_B_* = *D_T_ Q*^2^ its HWHM. Such a simple relation usually does not hold in the crowded medium of a macromolecule. The atoms diffuse, for instance, through a motion called translational jump-diffusion [23], where the HWHM does not increase linearly, but deviates from such behavior at higher *Q* values. The atoms perform oscillatory motions around their equilibrium positions for a time *τ*_0_. After that they diffuse for a time *τ*_1_ by continuous diffusion and the process is then repeated. The structure factor can be described by a single Lorentzian with the HWHM Γ_*JD*_ as:

(6) $$\Gamma_{JD}=\frac{D_{T}Q^{2}}{1+D_{T}Q^{2}\tau_{0}}$$

At small Q-values, the slope of Γ_*JD*_ gives again the translational diffusion coefficient *D_T_* and the relation reduces to Brownian’s law, i.e., Γ_*JD*_ = Γ_*B*_. At large Q-values, the HWHM tends to the asymptotic value Γ_∞_ = 1/*τ*_0_, *τ*_0_ being called the average residence time.

When the motions are further restricted, they can result in rotational diffusion within a confined space [24]. The structure factor describing such movements reads:

(7) $$S_{R}\left(Q,\omega \right)=A_{0}\left(\mathrm{QR}\right)\delta \left(\omega \right)+\frac{1}{\pi }\sum_{l=1}^{\infty }\left(2l+1\right)A_{l}\left(\mathrm{QR}\right)\frac{\Gamma_{l}}{\omega^{2}+\Gamma_{l}^{2}},$$

where the diffusing atoms are limited to a sphere of radius *R*, Γ_*l*_ = *D_R_ l*(*l* + 1), *D_R_* being the rotational diffusion coefficient. Volino and Dianoux [24] found an analytical expression for the EISF:

(8) $$A_{0}\left(QR\right)={\left[\frac{3j_{1}\left(QR\right)}{QR}\right]}^{2}$$

where *j*_1_(*x*) = sin *x*/*x*^2^ − cos *x*/*x* is the first-order spherical Bessel function. Bellissent-Funel et al. [25] expanded the model for the EISF by an immobile fraction *p*, where *p* denotes strongly bound protons. In that case, Equation (8) becomes:

(9) $$A_{0}\left(QR\right)=p+\left(1-p\right){\left[\frac{3j_{1}\left(QR\right)}{QR}\right]}^{2}$$

Although the jump diffusion and diffusion in a confined space models were first developed for motions in fluids, such models were also successfully applied for crowded media [26].

### 2.3. Molecular Dynamics Simulations

The crystallographic structure of mAChE dimers PDB ID 1J06 [27] was used as a source of the protein coordinates. The missing loop 258–264 was restored using Modeller 9.14 [28], and the missing Lys496(A) and Arg493(B) side chains were reconstructed by means of a VMD *psfgen* module. Hydrogen atoms were added to construct the hydrogen bonding network by using Reduce software [29]. Water molecules recognized in the crystal structure were included in the model system and other non-protein molecules were removed. To meet experimental protein concentrations in the solution and to avoid interactions of the proteins with its copies in PBC, two dimers (four protein molecules) were placed in solvation boxes. Clusters of sucrose molecules were built by means of VegaZZ software [30]. The total number of TIP3P water molecules and sucrose molecules added were adjusted to meet experimental concentrations. The number of molecules and sizes of the resulting systems are provided in Table S1 containing the electronic supplemental information (ESI). 

MD simulations were performed with the NAMD 2.11 program [31] with CHARMM36 force field [32,33] at the Lomonosov-2 Moscow State University supercomputer center [34]. Periodic boundary conditions and PME electrostatics were applied. The systems were maintained at a constant temperature 300 K for productive runs and under pressure 1 atm (NPT ensemble) by using Langevin dynamics and Nosé-Hoover barostat. The initially prepared system underwent a 3000-step minimization.

As sucrose molecules were added to the system as a regular grid, systems with sucrose were subjected to a long pre-equilibration to optimize the solvents with 1 fs time-steps. For this, the coordinates of protein atoms and surrounding 4 Å water shells were fixed. In the first time, systems were subjected to 20 ns MD simulations at 400 K, and in the second time cooled down to 300 K during 20 ns. Then 10 ns unconstrained runs were performed for final equilibration. A radial distribution function was used to control the equilibration of the sucrose solution (see ESI, Figure S1). Then, 1 μs productive MD-runs at 300 K with 2 fs time-steps were performed. The analysis of obtained MD trajectories was performed using VMD [35] software and custom scripts. For calculations of the number of hydrogen bonds, a distance of 3.2 Å and a 40° cutoff angle were used. The calculations of the number of atom–atom contacts involved hydrogen atoms. Mean square displacement values were calculated from MD trajectories with different lag times *τ* as follows:

(10) $$\langle u^{2}\left(\tau \right)\rangle ={\left[RMSF\left(\tau \right)\right]}^{2}=\langle {\left[\bar{r}\left(t+\tau \right)-\bar{r}\left(t\right)\right]}^{2}\rangle$$

where *RMSF* stands for root-mean square fluctuations.

Principle component analysis (PCA) and Cartesian PCA (cPCA) were performed using ProDy software [36], dihedral angle PCA (dPCA) was performed using Carma software [37]. cPCA was performed excluding the most mobile terminal residues 1–10 and 530–543. PCA methods allow us to determine and visualize the directions in space corresponding to the axes of highest mobility.

## 3. Results

The experiments were performed between June and August 2013 on the instruments IN6, IN13 and IN16 (DOI:10.5291/ILL-Data.8-04-707) and we repeated one measurement in August 2014 on IN13.

### 3.1. Incoherent Neutron Scattering

IN13 is a backscattering spectrometer giving access to averaged local motions within the time range of about 100 ps, e.g., it mainly enables the observation of sub-molecular motions of the amino acid side chains and small rotations at the protein’s surface.

**Figure 1:**
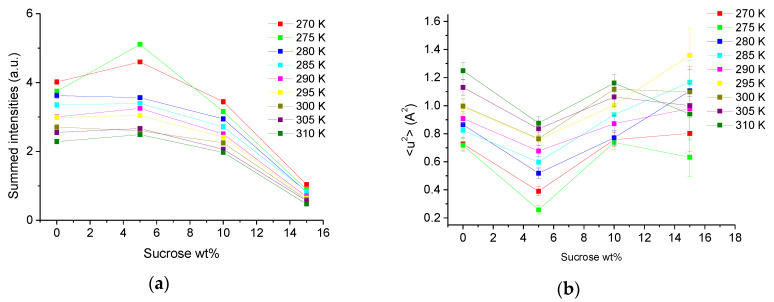
Samples measured as function of sucrose wt% for temperatures ranging from 270 to 310 K. (**a**) Summed elastic intensities. The error bars were calculated but are so small that they are within the symbols. (**b**) mean square displacement (MSD) extracted through Equation (2).

The four samples were scanned elastically within the temperature range from 270 to 310 K in steps of 5 K. After correction and normalization of the raw data, we first calculated the EINS of Equation (1) summed over all available *Q*-values. Such a quantity has smaller error bars due to the increased statistics and only depends on external parameters as temperature and the sucrose concentration. In Figure 1a, we present the summed intensities as function of wt% of sucrose at all measured temperatures. Higher temperature has the expected effect of lowering the intensities. In contrast, increased intensity depicts slower dynamics as more neutrons are scattered in a way to stay within the instrumental time window. All curves show a non-linear dependence on the sucrose concentration, with a maximum of intensity around 5 wt%. At 10 wt%, the intensities drop significantly, but present a pronounced dependence on temperature. In contrast, at 15 wt% all points seem to converge. We further used Equation (2) to extract the MSD of the same data and to plot them also as function of sucrose concentration (see Figure 1b). The MSD are mainly inversely proportional to the square of the summed intensities [38], so here we find a minimum at around 5 wt%. As the data have lower statistics, the errors are bigger and the variations higher, especially at 15 wt% where the protein quantity and therefore the data quality was below the other curves.

**Figure 2:**
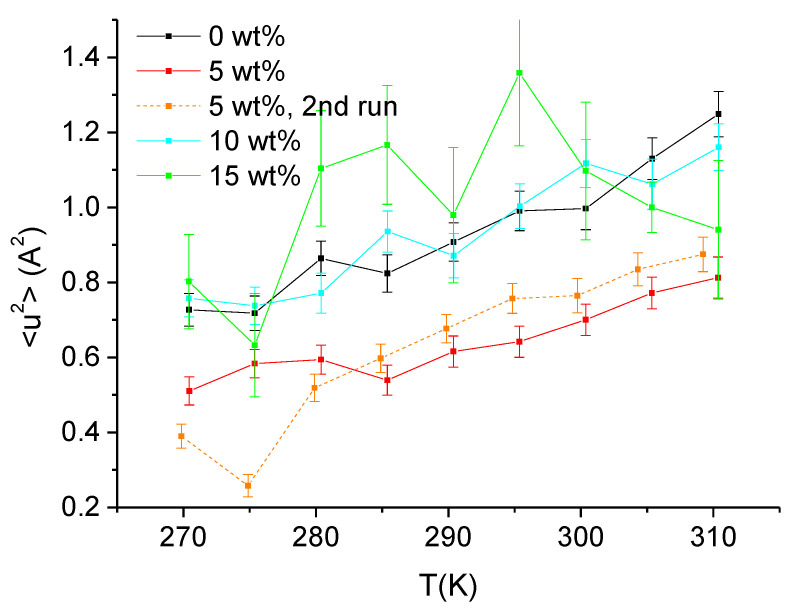
MSD extracted through Equation (2) from data taken on IN13 as function of temperature. The black curve corresponds to the sample with no sucrose, the red and orange curves to the sample with 5 wt% sucrose, the cyan curve to 10 wt% sucrose, and the green curve to 15 wt% sucrose.

Figure 2 presents the same MSD extracted from IN13 data now as function of the temperature for different concentrations of sucrose. All samples follow a linear increase in temperature except the sample at the highest sucrose concentration, which presents rather high fluctuations due to low data quality. As the behavior at 5 wt% sucrose was unexpected, we reopened the sample holder, dried, and rehydrated the sample and repeated the measurement. The result is shown in the orange dashed curve, close to the red curve, except at low temperature, where we saw probably the melting of heavy water around 277 K the second time.

IN6 has a time window of about 20 ps and gives access to smaller and faster motions, which are for instance directly influenced by the presence of water molecules. Here, we measured EINS and QENS spectra. Globally, the MSD are lower compared to those obtained on IN13, what is explained by the smaller time window of IN6, but followed the same trends (see ESI, Figure S2) confirming further the non-linear behavior around 5 wt% sucrose.

**Figure 3:**
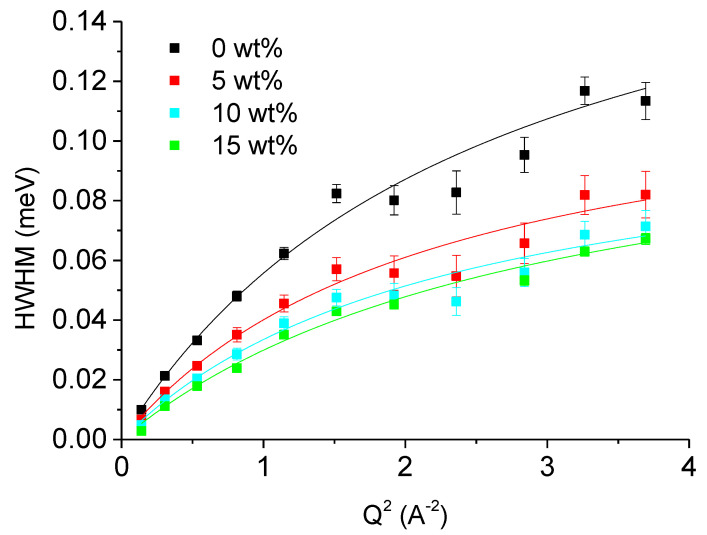
Half width half maximum (HWHM) Γ of the one Lorentzian curve used to fit the Quasi-elastic neutron scattering (QENS) data of D_2_O in presence or not of sucrose taken on IN6 as function of Q^2^. The black curve corresponds to no sucrose, the red curve to 5 wt% sucrose and the cyan curve to 10 wt% sucrose. The lines are fits using the jump-diffusion model Equation (6).

QENS data were collected at 310 K, the physiological temperature of living organisms, and analyzed with the formalism described above (see Equations (3) and (4)). First, the buffer (D_2_O in presence or not of sucrose) curves were fitted with two Lorentzian functions and their HWHM extracted. Water molecules can simultaneously undergo translational and rotational motions and, indeed, we obtained this type of motions. Figure 3 shows the HWHM of the first Lorentzian curve together with fits according to the jump-diffusion model described in Equation (6). The systematic dip around 2.5 Å^−2^ can be ascribed to de Gennes narrowing consequent to coherent scattering. The second Lorentzian was about 5–6 times larger, corresponding to faster motions, and corresponded to rotational motions as identified by the absence of a Q-dependence (data not shown).

All four samples present the typical Q-dependence of water at ambient temperature [39] and in the presence of co-solutes, corresponding to random-jump diffusion and rotations. In particular, our curves compare very well to those presented by Grimaldo et al. [40] for D_2_O measured also on IN6 at the ILL, Grenoble. The addition of sucrose had the expected effect: viscosity was increased and thus the translational diffusion coefficient D_T_ decreased from 1.17 (5) to 0.89 (4), 0.73 (4) and 0.61 (3) 10^−5^ cm^2^/s with the increasing sucrose concentration. The residence times τ_0_ obtained for the four cases were 3.3 (3), 5.2 (5), 5.9 (6), and 5.5 (5) ps, respectively. In comparison, pure bulk H_2_O water has the characteristics *D*_T_ = (2.5 ± 0.1) 10^−5^ cm^2^/s; and *τ*_0_ = 1.1 ps [25], which represent a higher and lower limit for these parameters. The self-diffusion in D_2_O is moreover slightly below that in H_2_O [41].

**Figure 4:**
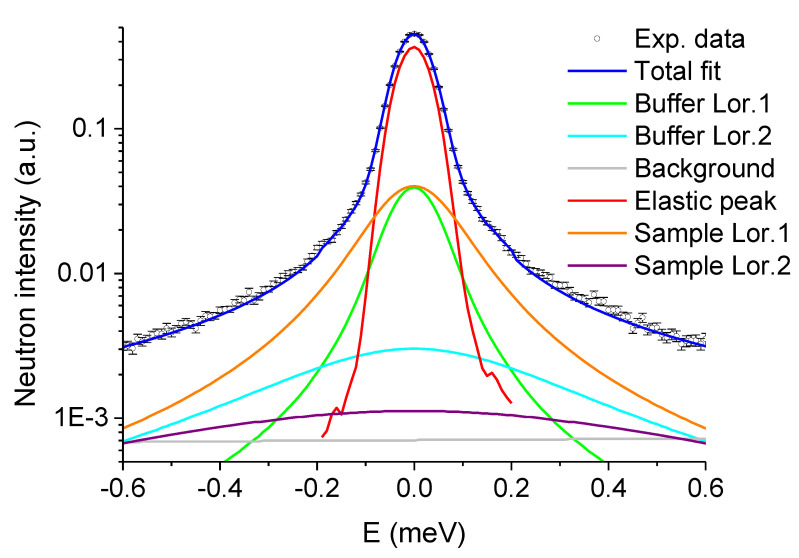
Logarithmic plot of an example of a fit of experimental QENS data of sample in the presence of 5 wt% sucrose for Q = 0.9 Å^−1^ at 310 K. As described above, two Lorentzian curves were used for the buffer, two Lorentzian curves for the sample, a delta function to account for the elastic peak and a straight line for the background, resulting in a fit quality factor χ^2^ = 1.38.

For the analysis of QENS data of the complete samples, we fixed then the HWHM of the two Lorentzians attributed to the buffer as well as the ratio of the absolute intensities between them, but let their absolute values vary freely together. Then, two Lorentzians were added to account for the sample. Figure 4 presents an example of such a fit of the experimental data with one elastic peak, four Lorentzian curves and a straight line to account for the background, which gives very good fit results.

**Figure 5:**
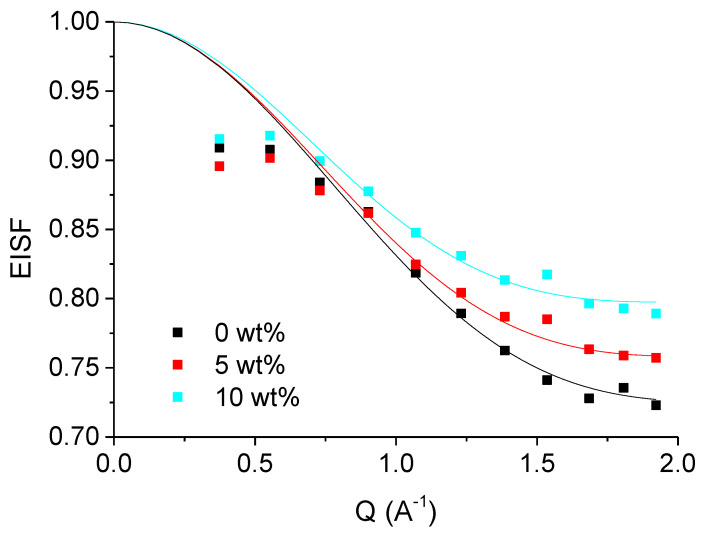
Elastic incoherent structure factor (EISF) of the samples with no sucrose (black curve), with 5 wt% sucrose (red curve) and with 10 wt% (cyan curve) sucrose as function of Q.

We determined first the EISF A_0_ (Q) for the three samples with no, 5 and 10 wt% sucrose (see Figure 5). According to the diffusion-in-a-sphere model and Equation (9), the value of the EISF at highest Q is representative for the proportion p of particles, e.g., H nuclei and molecular subgroups to which they are bound, seen as immobile within the instrumental time resolution. R is representative of the radius of the sphere delimiting the particles’ motions. Both quantities are progressively increasing with sucrose concentration (see Table 2), although the radius is mainly the same for all samples within error bars. Due to the increasing viscosity of the solution in the presence of sucrose, more particles are slowed down at higher sucrose concentration.

**Figure 6:**
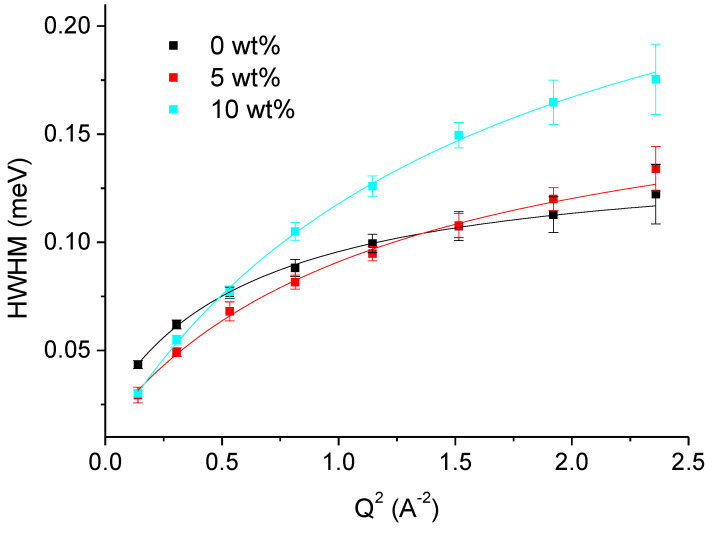
HWHM Γ of the narrower Lorentzian curve used to fit the QENS data of mAChE in the presence or not of sucrose taken on IN6 as function of Q^2^. The black curve corresponds to no sucrose, the red curve to 5 wt% sucrose and the cyan curve to 10 wt% sucrose. The lines are fits using the jump-diffusion model Equation (6).

**Figure 7:**
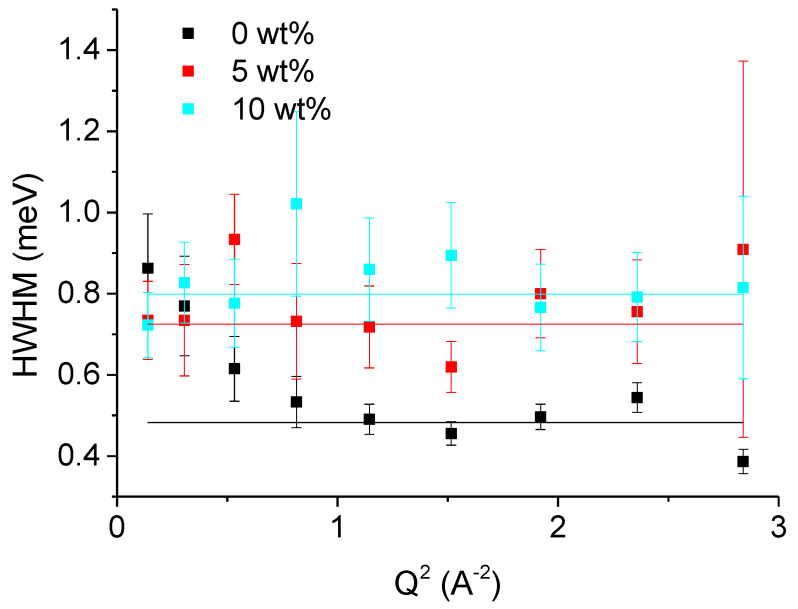
HWHM Γ of the broader Lorentzian curve used to fit the QENS data of mAChE in the presence or not of sucrose taken on IN6 as function of Q^2^. The black curve corresponds to no sucrose, the red curve to 5 wt% sucrose and the cyan curve to 10 wt% sucrose. The lines are fits with a constant value corresponding to rotational diffusion.

Further, we determined the HWHM of the Lorentzian curves of the samples (see Figure 6 and Figure 7), plotted them as function of Q^2^ and fitted them with one of the models.

Figure 6 presents the HWHM Γ of the narrower Lorentzian function, corresponding to larger movements. We reiterate here that we are probing the internal motional diffusion of small molecular subgroups rather than global diffusion of the protein in solution, which is beyond the scope of the instrumental resolution of IN6. In this case, the tendencies are inverted with the curve at 10 wt% being clearly above the two others, which are more similar in absolute values. The Q-dependence of the HWHM still resembles jump-diffusional motions, but the slopes and curvatures differ significantly for the three samples and we notices again the non-linear behavior. The translational diffusion coefficient D_T_, corresponding to the slope at small *Q*-values, varies from (2.9 ± 0.4) 10^−5^ cm^2^/s without sucrose to (2.1 ± 0.3) 10^−5^ cm^2^/s at 5 wt% and to (2.7 ± 0.2) 10^−5^ cm^2^/s at 10 wt% sucrose. The residence times τ_0_ obtained for the three cases were now (5.5 ± 0.2), (3.8 ± 0.2) and (2.3 ± 0.1) ps, respectively.

Figure 7 shows the HWHM of the broader Lorentzian curve, used to fit the QENS data. The obtained values are tainted with bigger error bars and do not show a systematic dependence on Q. These smaller molecular motions can be attributed to rotational diffusion, which does not depend on Q. The HWHM Γ is related to the rotational diffusion coefficient Γ_R_ = 2D_R_ (see Equation (7)). Here, the effect of sucrose has an opposite effect: an increase of the rotational diffusional coefficient from 0 to 10 wt% yielding the values for D_R_ of (0.24 ± 0.02) meV, (0.36 ± 0.02) meV, and (0.4 ± 0.01) meV, respectively.

Finally, IN16 is the instrument with the highest energy resolution which thus allows us to probe motions up to about 1 ns. Such a time window might provide us with further information on local motions, however, it also includes motions of subgroups and domains. We recorded EINS data from 275 to 312 K and plotted the summed intensities (see ESI, Figure S3a) and MSD (see ESI, Figure S3b). At these longer times, the differences are becoming even more striking, but again the hierarchy between the samples is the same.

### 3.2. MD Simulations

**Figure 8:**
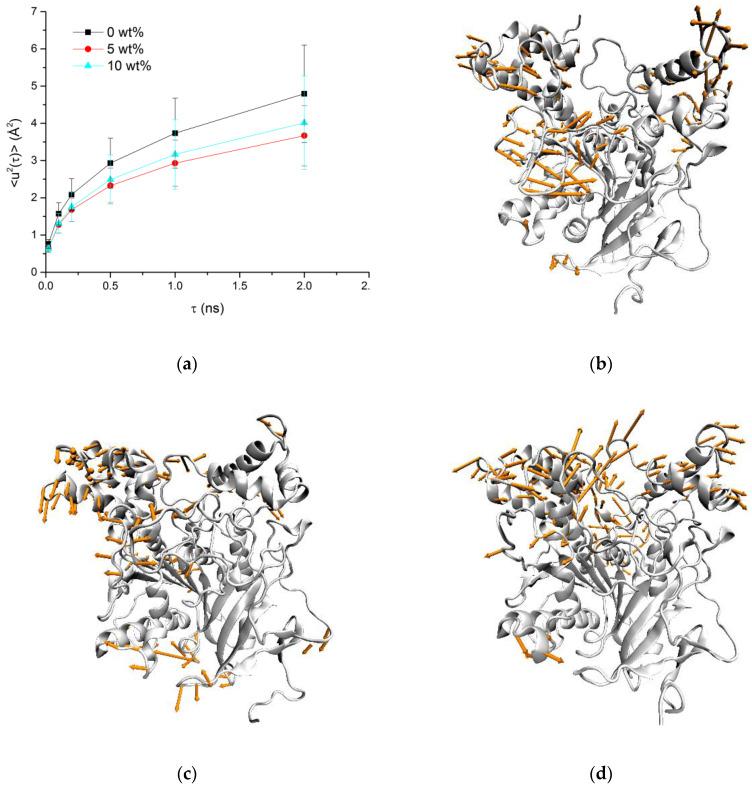
(**a**) MSD vs. τ for different sucrose concentrations. The first cPCA modes with the greatest variance and for mAChE in 0 wt% (**b**), 5 wt% (**c**) and 10 wt% (**d**) sucrose solutions.

Mean square displacement values $\langle u^{2}\rangle$ calculated for protein over MD trajectories with different time windows (τ) showed nonlinear patterns, as it was observed experimentally: a decrease in mobility at 5 wt% sucrose, compared to 0 wt%, and an increase at 10 wt% sucrose solution (Figure 8a). This effect becomes more pronounced with the increase of τ. The per-residue RMSF distribution is ambivalent, showing increased and decreased mobility of some areas in all systems (see ESI, Figure S4).

PCA of the MD trajectories allows to separate low frequency motions and to visualize differences in mAChE mobility (Figure 8b–d). cPCA shows that in the 0 wt% sucrose sample, the most mobile segments are peripheral loops, including loop 258–264, not resolved in X-ray, and Ω-loop. In the 5 wt% sucrose solution, these movements are dampened (amplitude decreased). However, Ω-loop movements are accompanied by acyl loop movements due to the binding of sucrose molecules at the gorge entrance. In the case of 10 wt% sucrose, movements of most of α-helixes and loops constituting the gorge walls (Ω-loop and acyl loop) have considerably increased their variance compared to movements in more diluted solutions, while β-sheets spanning through the protein are not affected. Other representations including network views of the cPCA results are shown in the ESI, Figure S5. dPCA free energy landscapes have distinctive local basins in case of 0 wt% and 10 wt% sucrose solutions, while for 5 wt% free energy landscape domains are more connected (ESI Figure S5).

**Figure 9:**
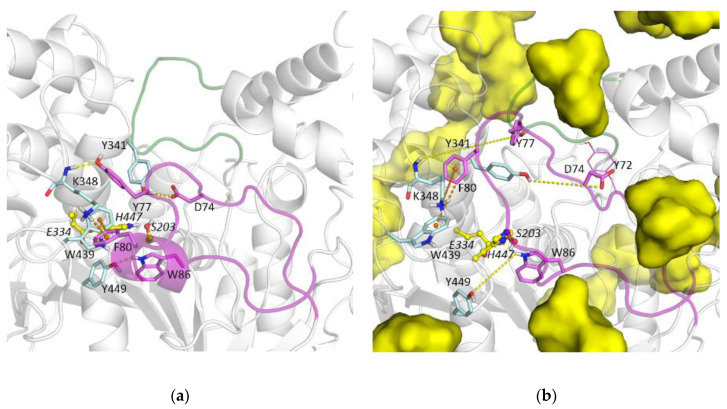
Ω-loop constituting the wall of the mAChE active site gorge (purple) and its interaction with the adjacent part of the protein, yellow dashes show hydrogen bonds, orange dashes show π-π and π-cation interactions, sucrose molecules are shown with yellow surface representation. (**a**) X-ray structure, (**b**) snapshot after 1 μs MD trajectory of the 10 wt% solution system, connections between atoms shown in panel (**a**) as interactions are maintained. Acyl loop forming the other wall of the gorge is shown in green.

mAChE conformational states corresponding to the deepest basins seen for the 10 wt% solution reflect movements of the Ω-loop, induced by interactions with the sucrose molecule. Figure 9a shows the interactions of the Ω-loop with the adjacent part of the protein. They are few: hydrogen bonds between Asp74 and Tyr341, Tyr77, and Lys348 main chain, Trp86 and Tyr449; π-cation interaction between Phe80 and Lys348 and T-stacking interaction between Phe80 and Trp439. Panel B shows that all these interactions are disrupted during MD simulation of mAChE in 10 wt% sucrose solution. Yet, the Ω-loop is only partially detached through intermittent interactions, catalytic triad remains operative, and Trp86 forms cation-binding site. Furthermore, the snapshot shows a typical situation for MD simulations of ChEs in sucrose solutions: the sucrose molecule at the entrance of the gorge interacting with the Ω-loop and acyl loop (Figure 9b and ESI Figure S6). This results in the increased mobility of the acyl loop (Figure 8) and correlated motions between the Ω-loop and acyl loop residues (ESI Figure S6).

Indeed, despite the observed increase in mobility of Ω-loop and acyl loop, there is no sign of partial unfolding, induced by the increase in sucrose concentration, or change in the gyration radius *R*_g_, or in the solvent accessible surface (SASA) (Table 3).

The number of water molecules inside the active site gorge is decreasing in the 5 wt% system due to osmotic stress [42]. The depletion of water molecules increases the mobility of the gorge residues (ESI, Figure S4b), but cPCA shows that these are high frequency motions and the catalytic triad remains operative. In the 10 wt% sample, this number is increased due to the instability induced by sucrose molecules on the Ω-loop and acyl loop. It opens new windows including a so-called backdoor [43] and side-door [44] for the influx of water molecules and reduces the accuracy of estimated water molecules inside the gorge (ESI Figure S6).

The averaged numbers of hydrogen bonds and atom–atom contacts have high variances; however, some tendencies could be noticed. Between the 0 wt% and 5 wt% sucrose systems, the number of protein–water interatomic contacts and hydrogen bonds slightly decreases. At the same time, the sum of the numbers of protein–water and protein–sucrose interatomic contacts and hydrogen bonds for the 5 wt% system is higher than the numbers of protein–water interatomic contacts and hydrogen bonds for the 0 wt% system. It can be interpreted as follows, when approaching the protein surface, the sucrose molecules rather interact with it through water molecules than to displace them. In addition, this could be the sign of a stiffer surrounding of the protein.

Comparing the 5 wt% and 10 wt% sucrose systems, the total number of hydrogen bonds and atom–atom interactions decreases as the percentage of sucrose is increased, and the replacement of water with sucrose molecules is more pronounced. Direct interactions of sucrose molecules with the protein surface does not lead to a significant decrease in protein intramolecular hydrogen bonding.

**Figure 10:**
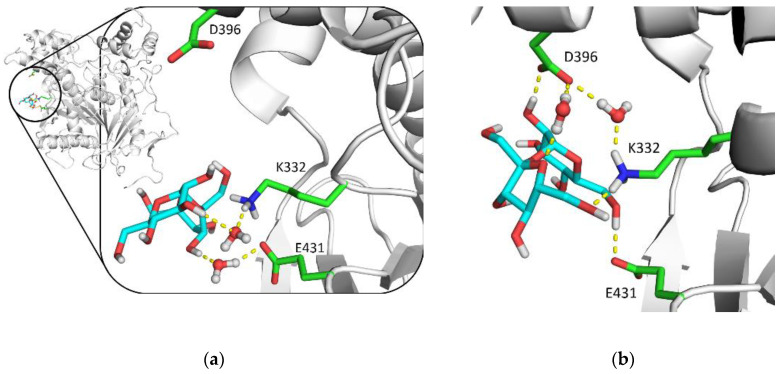
5 wt% system (**a**) and 10 wt% system (**b**) snapshots after 1 μs MD trajectory showing the interaction of sucrose molecules with the same residues.

These differences between the nature of interactions between sucrose molecules and the protein in the 5 wt% and 10 wt% sucrose systems are illustrated by the example in Figure 10. In the 5 wt% sucrose system, the sucrose molecule interacts with mAChE residues through water molecules, on the contrary, in the 10 wt% sucrose system some interactions are through water molecules, others are direct. The salt bridge between Lys332 and Glu431 is broken, but the distant residue Asp396 is brought closer. This effect is similar to the one described above, the crosstalk between Ω-loop and acyl loop mediated by sucrose molecules. It can be seen as new contacts (correlated motions) in the network view of cPCA results (ESI Figure S5).

## 4. Discussion

The role of viscosity on the sub-ns molecular dynamics of proteins has already been investigated in the past by means of incoherent neutron scattering and a clear impact was determined [45,46,47] of the presence of glycerol or glucose. As expected, co-solutes decreased the MSD of lysozyme, a small globular protein, in these cases above 150 K, but prevented the freezing even at low temperature. We were able to observe here similar behavior in the case of pure D_2_O buffer, which is more and more slowed down under the effect of sucrose (see Figure 3). The corresponding translational diffusion coefficients are all below the value known from the literature for bulk H_2_O [39]. In agreement with these findings, the residence times increased. The latter effect is indicative for a stronger confinement effect [48]. The result found for the EISF of our samples points towards the same direction, as it increases at high Q values with increasing concentration of sucrose, meaning that less particles participate to the motions resolved by the instrument IN6 in the presence of the sugar although the radius of confinement is almost constant.

On the contrary, the results concerning the summed intensities and MSD extracted from three different spectrometers with increasing time window, IN6, IN13, and IN16, tell us a different story. As expected, the MSD increase with the time window, as more and more movements become visible at longer scales. The same findings hold for MD simulations as shown in Figure 8a. The MSD also increase with temperature, as the additional thermal energy permits larger amplitudes. However, the MSD equally increase with the concentration of sucrose, except for the sample at 5 wt% which presents a strong non-linear behavior. In particular, for IN6 we observe a cross-over of the curves corresponding to 0 and 5 wt% sucrose at around 290 K. Obviously, at this short time scale of 20 ps, the corresponding motions set in only at higher temperatures.

Several explanations are possible for such insights and only the MD simulations executed in parallel on the same kind of samples permitted to verify and better understand these effects:

1. When sucrose molecules are located at the entrance of the gorge, the gorge acts as semi-permeable membrane, and water can get out, but not enter due to the osmotic effect. Such an effect was previously observed with human BChE [42]. Moreover, sucrose molecules cannot enter due to their volume. At 0 wt% sucrose, there is only water inside and outside the cavity. The simulations allowed us to see that at 5 wt% sucrose, the motion of the protein’s outer part is dampened, but inside the gorge motion is increased due to water depletion. Finally, at 10 wt% sucrose structural modifications seem to occur in such a way that the sugar molecules form a layer at the protein’s surface and protect the water layer, which permits a higher flexibility of the enzyme. These results are reflected in the neutron scattering data through a higher stability at 5 wt% sucrose and higher mobility at 10 wt%.
2. Sugars have a small binding affinity for cholinesterases. It seems that the enzymes become more flexible in the presence of sugar. This increases both the conformational entropy and the affinity to bind, so that water molecules bound on the surface can be replaced by sugar [49].
3. Similar effects were reported earlier by Cicerone et al. [50] and Curtis et al. [51], showing that a binary glycerol–trehalose glassy matrix can stabilize a protein at a particular mass fraction of glycerol. The minimum in flexibility of the protein coincides with the lowest flexibility of trehalose in the binary glass. The authors have shown that the stabilization of the protein is due to the suppression of local fast motions in both the host matrix and the protein, although much of the important dynamics of the protein may not be coupled with viscosity of the host fluid.

QENS measurements show furthermore that not only molecular dynamics are reduced around 5 wt% of sucrose, but that the nature of the movements is also changed. Whereas the curves at 0 and 10 wt% of sucrose for the HWHM resemble to jump-diffusion motions, the curve at the intermediate sucrose concentration looks stiffer. This agrees with the fact that the modes at the protein’s surface are dampened according to the simulations. The translational diffusion coefficients are indeed similar at 0 and 10 wt% but decreased in between. A re-structuration at the protein’s surface and a strong coupling between the water layer and the sucrose matrix seems thus likely.

MD simulations are in good agreement with the experimental data and confirm the outstanding role of the interaction between water molecules, the sugar matrix and the protein surface. This allows us to understand the dynamics of the protein, e.g., the nonlinear behavior increasing over the time window. The analysis of MD trajectories, though partly speculative where values have high variances, allows us to suggest the following explanation: with the addition of sucrose at 5 wt%, the protein’s nearest environment becomes more populated or stiff, reducing protein mobility as seen with neutron scattering. With an increase of the concentration, sucrose molecules replace more water molecules and have more direct interactions with the protein’s surface. At 5 wt%, the water shell protects the protein from sticky sucrose molecules, at 10 wt% this shell is depleted and this disturbs protein dynamics. Sucrose molecules penetrating the protein surface disrupt some of the intra-protein contacts, and induce new ones, creating new pathways for correlated motions. This effect is amplified by interactions between sucrose molecules, more frequent in more concentrated solutions.

It remains uncontested that the study of a protein in the presence of high sugar concentrations is not directly comparable to its native form. However, our observations shed light on the mechanisms of water fluxes into and out of the gorge, including the active site. Our investigations permitted us to conclude on complex dynamics of the Ω- and acyl loop. Other parts of the enzyme or polypeptide segments may also display peculiar dynamics. This opens new windows for understanding the extraordinary catalytic power of cholinesterases. In particular, the influx of water molecules across a backdoor could be addressed by MD simulations in the presence and absence of sucrose. The functionality of a backdoor as it was suggested [43] remains a puzzling issue. In the present case, simulations allowed to get information about the number of water molecules inside the gorge for the different samples. In contrast to what we expected (e.g., a continuous decrease of water molecules due to the osmotic effect and a concomitant shrinking of the gorge) maybe up to a collapse, we observed a much more subtle interplay between the different components and an increase in protein mobility at the highest sucrose concentration. The latter can be ascribed to the instability induced by sucrose molecules on Ω-loop and acyl loop, which finally results in conformational perturbation of the gorge.

## 5. Conclusions

In the present work, we intended to investigate the dynamics of the enzyme mAChE around its narrow gorge and of the water molecules, which move quickly into and out of the gorge. As the per-deuteration of mAChE is not possible so far, we opted for a study in D_2_O solution and in the presence of increasing concentration of deuterated sucrose. The sugar exerts osmotic pressure on the water molecules in the gorge permitting to study these movements in more detail. Surprisingly, we found experimentally a non-linear behavior in the dynamics of the samples with increasing sucrose concentration, inducing first a slowing down of the dynamics at 5 wt% and then again a raise. Moreover, the nature of the dynamics was also found different at the minimum of mobility. Extensive MD simulations allowed us to study in detail the interactions of the different components leading to such behavior. Sucrose molecules interact with the surface of the protein and the entrance of the gorge at a lower concentration through the water layer, damping the motions at the surface, but increasing them inside the gorge. When increasing the sucrose concentration more, the sucrose molecules replace some of the water molecules at the surface, permitting again to more water molecules to enter the gorge and opening simultaneously new pathways, among them the hypothesized backdoor to the gorge. These multiple mutual influences and interactions permit us to explain very satisfactorily the experimental findings.

## Figures and Tables

**Table 1:** Sample masses used for the experiments.

(*w*/*w*)% Sucrose	Mass of mAChE (mg)	Mass of Sucrose (mg)	Mass of D_2_O (mg)	Final Sample Mass (mg) after Rehydration
0	100	0	208	308
5	84	17.8	228	339.8
10	70	30.8	214	314.8
15	56	46.4	296	398.4

**Table 2:** Proportion p of particles seen as immobile and radius R of the sphere.

(*w*/*w*)% Sucrose	Proportion *p*	Radius R (Å)
0	72.6 (5)%	2.11 (6)
5	75.8 (3)%	2.24 (6)
10	79.8 (6)%	2.3 (1)

**Table 3:** Averaged parameters derived from 1 μs MD trajectories (±SD).

	Water	5 wt% Sucrose	10 wt% Sucrose
SASA, Å^2^	23,991 ± 367	23,934 ± 273	24,047 ± 297
*R*_g_, Å	23.0 ± 0.1	23.0 ± 0.1	23.0 ± 0.1
Number of water molecules inside the gorge	47 ± 4	31 ± 4	43 ± 5
Number of hydrogen bonds			
Protein–water	270 ± 10	268 ± 10	256 ± 10
Protein–sucrose		8 ± 3	12 ± 4
Intra-protein	317 ± 9	317 ± 10	314 ± 10
Number of atom–atom contacts (2 Å cutoff)			
Protein–water	726 ± 21	713 ± 22	687 ± 20
Protein–sucrose		19 ± 6	33 ± 7
Sum		732 ± 21	720 ± 20

## Supplementary Materials

The following are available online at https://www.mdpi.com/2218-273X/10/12/1664/s1, Table S1: Sample proportions used for the simulations. Figure S1: MSD extracted through Equation (2) from data taken on IN6 as function of temperature. Figure S2: Summed intensities (a) and MSD (b) extracted through Equation (2) from data taken on IN16 as function of temperature. Figure S3: Protein surface shown for the snapshot in Figure 10B. Figure S4: cPCA results presented as network view and with color-coded backbone mobility.
