# Supplementary material for: Impact of Sucrose as Osmolyte on Molecular Dynamics of Mouse Acetylcholinesterase
Source: Biomolecules. 2020 Dec 12;10(12):1664. doi: 10.3390/biom10121664 (PMC7763276; doi:10.3390/biom10121664)
Supplement: Supplementary file 1 [file biomolecules-10-01664-s001.pdf]

Supplemental information

Impact of osmotic stress on molecular dynamics of mouse acetylcholinesterase

Sofya Lushchekina [1], Gaetan Inidjel [2,3], Nicolas Martinez [2,3], Patrick Masson [4], Marie Trovaslet-Leroy [5], Florian Nachon [5], Michael Marek Koza [2], Tilo Seydel [2], and Judith Peters [2,3]*

Table S1. Sample proportions used for the simulations.

(sucrose/total)*100%	mAChE/total, %	H_2O/total, %	Total number of atoms	Size, Å×Å×Å
0.00	31.4	67.53	119 951	107 x 78 x 137
5.40 (5.38)	25.53	69.13	147446	115 x 82 x 148
9.78 (9.53)	21.66	67.98	173 405	116 x 93 x 151

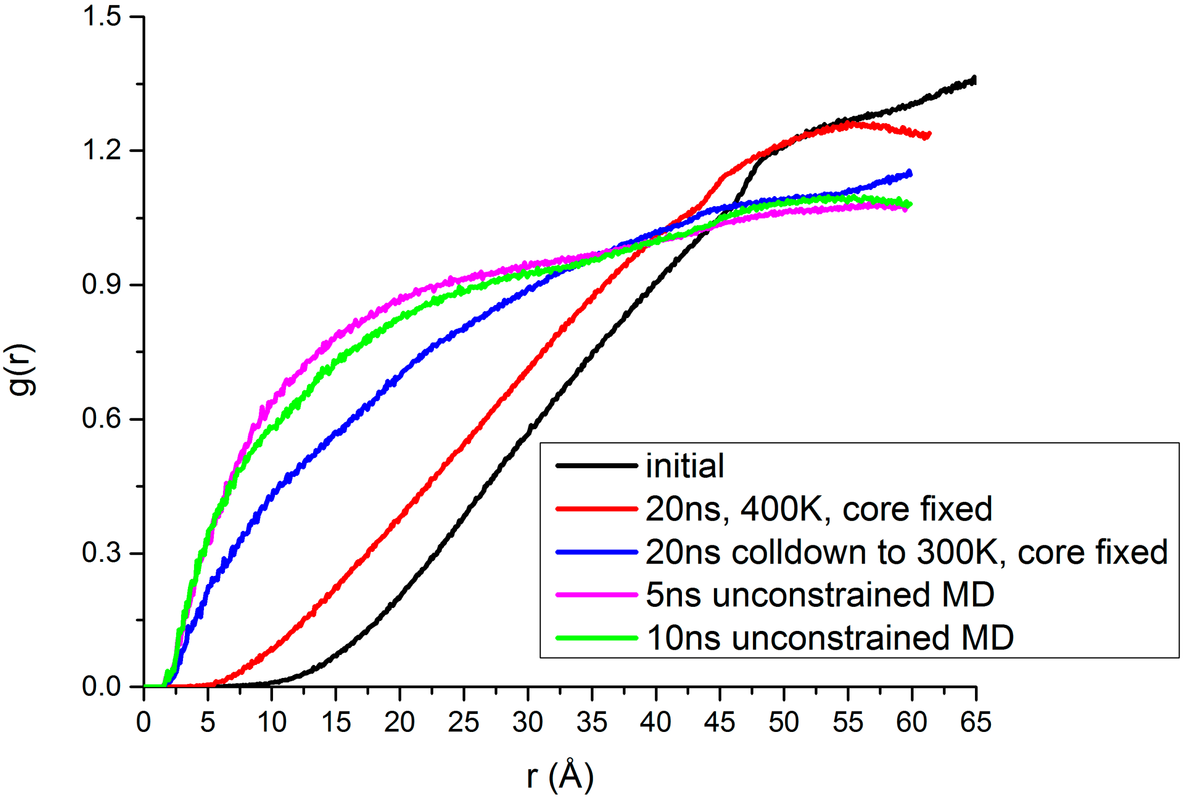

Figure S1. The radial distribution function (RDF) for sucrose molecules around mAChE molecules (10 wt%). Black line: the initial system with sucrose molecules added as regular grid. Red line: after 20 ns MD run at 400 K with the atomic coordinates of the proteins and the surrounding 4Å water shells fixed. Blue line: after 20 ns MD run with a gradual cooling from 400 K to 300 K with the atomic coordinates of proteins and the surrounding 4Å water shells fixed. Magenta line: after 5 ns unconstrained MD run. Green line: after 10 ns unconstrained MD run. Since the RDF for the last two cases are very close, the solution was considered as equilibrated.

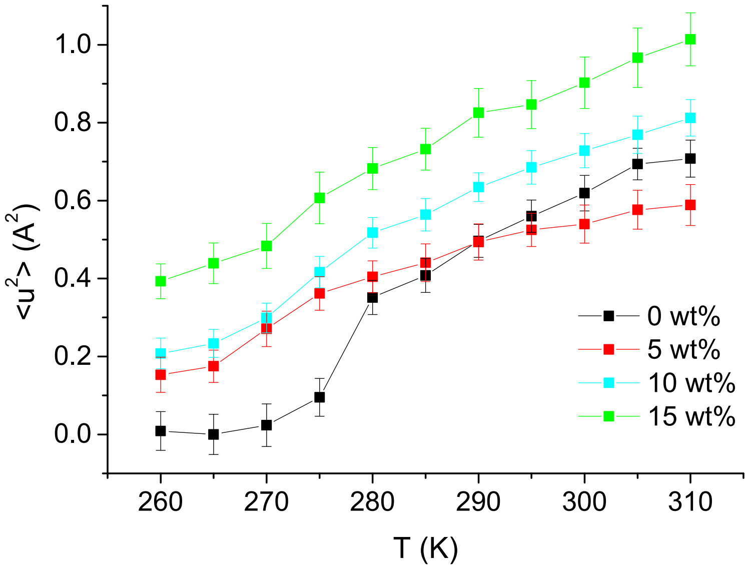

Figure S2: MSD extracted through eq. (2) from data taken on IN6 as function of temperature. The black curve corresponds to the sample with no sucrose, the red curve to the sample with 5 wt% sucrose, the cyan curve to 10 wt% sucrose and the green curve to 15 wt% sucrose.

The 0 wt% curve is here below the 5 wt% curve up to 280 K, likely due to freezing of water in the pure sample in contrary to the other samples, as sucrose has a protective effect against freezing. Around 285 K there is a cross-over and again the 5 wt% curve stays below the 0 wt% curve. As the neutron flux is higher on IN6 than on IN13, the statistics is also better for the sample with the highest sucrose concentration, which shows a curve very similar to the 10 wt% sample, but slightly upshifted.

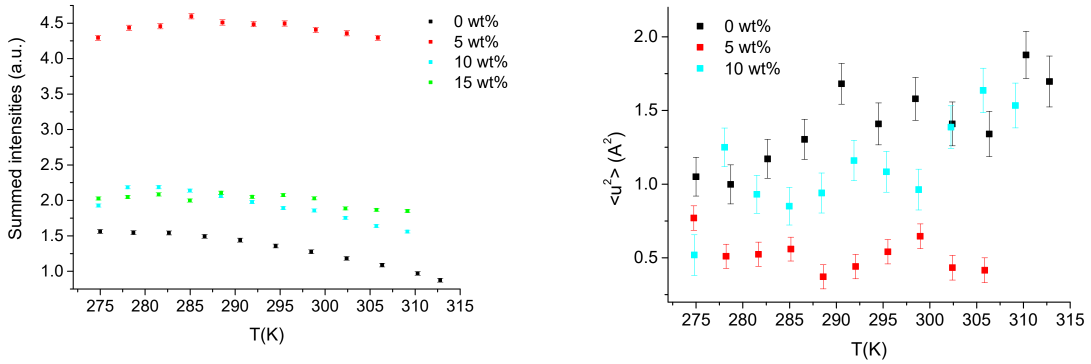

(**a**) (**b**)

Figure S3: Summed intensities (a) and MSD (b) extracted through eq. (2) from data taken on IN16 as function of temperature. The black curve corresponds to no sucrose, the red curve to 5 wt% sucrose, the cyan curve to 10 wt%-sucrose and the green curve to 15 wt% sucrose.

For 15 wt% sucrose, we show only the summed intensities, as the statistics is again not good enough to reasonably extract the MSD. The difference between the samples becomes striking at the longer time scales. The sample with 5 wt% sucrose has clearly the lowest dynamics, is therefore very stable, and does not change much with temperature. In contrary, the samples with no or 10 wt%-sucrose result in rather similar MSD, which show the typical increase with temperature.

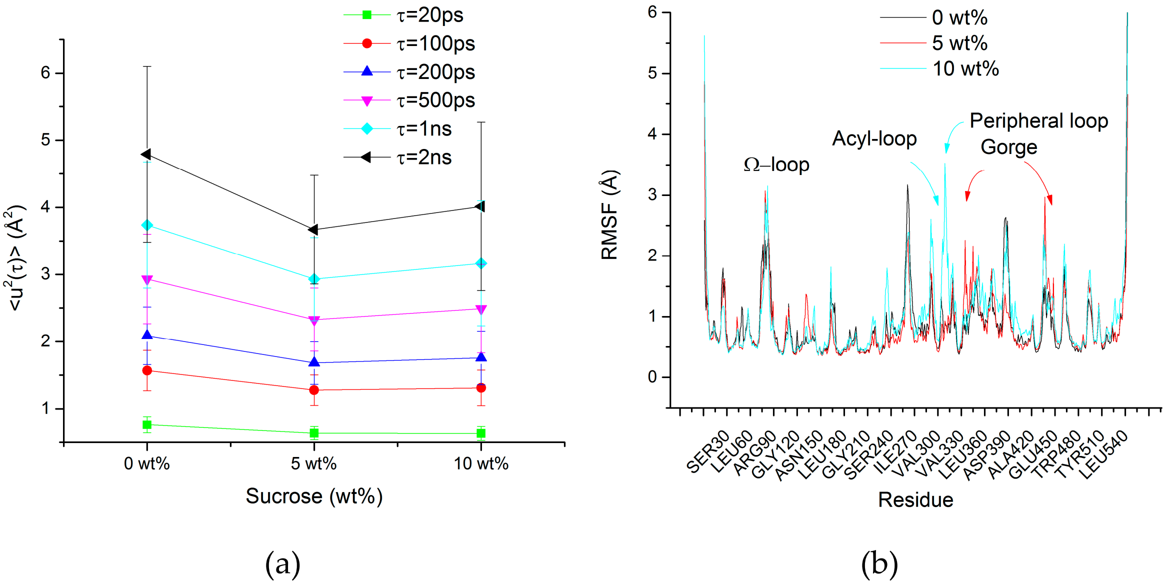

Figure S4. (a) MSD *vs* sucrose concentration for different τ values; (b) per-residue RMSF values (τ = 200 ps).

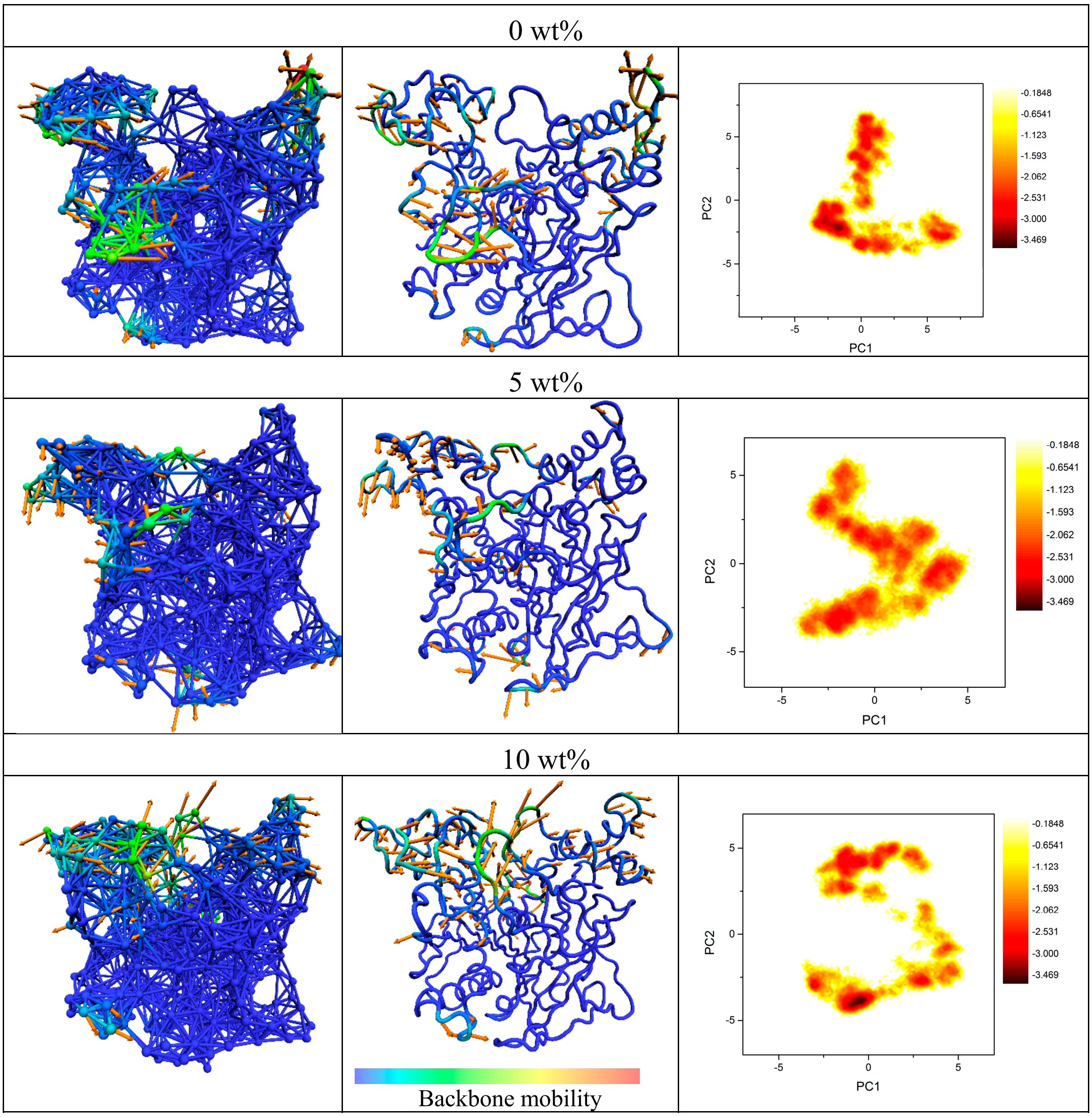

Figure S5. cPCA results presented as network view (left column) and with color-coded backbone mobility (middle column). dPCA free energy landscapes along the first and second principle components (right column). Free energy plots have the same scale in kcal/mol.

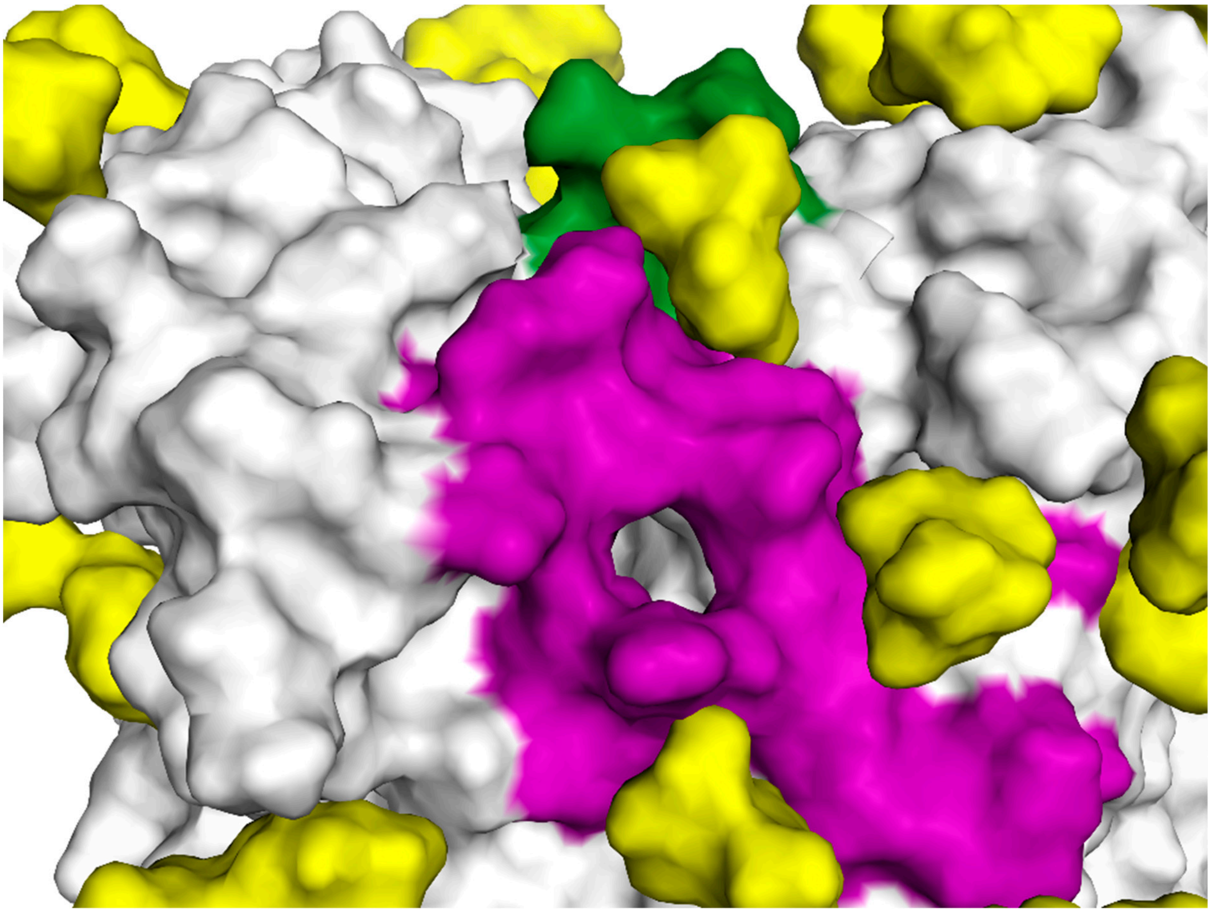

Figure S6. Protein surface shown for the snapshot in Figure 9b. A sucrose molecule bridging the Ω-loop (violet) and an acyl loop (green) are shown. The hole in the Ω-loop is referred in the literature as "side door" [1-4].

References

1. Van Belle, D.; De Maria, L.; Iurcu, G.; Wodak, S.J. Pathways of ligand clearance in acetylcholinesterase by multiple copy sampling. *J. Mol. Biol.* **2000**, *298*, 705-726, doi:10.1006/jmbi.2000.3698.
2. Tai, K.; Shen, T.; Henchman, R.H.; Bourne, Y.; Marchot, P.; McCammon, J.A. Mechanism of acetylcholinesterase inhibition by fasciculin: a 5-ns molecular dynamics simulation. *J. Am. Chem. Soc.* **2002**, *124*, 6153-6161, doi:10.1021/ja017310h.
3. Bui, J.M.; Tai, K.; McCammon, J.A. Acetylcholinesterase: enhanced fluctuations and alternative routes to the active site in the complex with fasciculin-2. *J. Am. Chem. Soc.* **2004**, *126*, 7198-7205, doi:10.1021/ja0485715.
4. Wiesner, J.; Kriz, Z.; Kuca, K.; Jun, D.; Koca, J. Influence of the acetylcholinesterase active site protonation on omega loop and active site dynamics. *J. Biomol. Struct. Dyn.* **2010**, *28*, 393-403, doi:10.1080/07391102.2010.10507368.
